# Assessing the Effect of an Integrated Control Strategy for Schistosomiasis Japonica Emphasizing Bovines in a Marshland Area of Hubei Province, China: A Cluster Randomized Trial

**DOI:** 10.1371/journal.pntd.0002122

**Published:** 2013-03-14

**Authors:** Xi-Cheng Hong, Xing-Jian Xu, Xi Chen, Yue-Sheng Li, Chuan-Hua Yu, Yi Yuan, Yan-Yan Chen, Ren-Dong Li, Juan Qiu, Zong-Chuan Liu, Ping Yi, Guang-Hui Ren, Hong-Bin He

**Affiliations:** 1 School of Public Health & Global Health Institute, Wuhan University, Wuhan, Hubei, People's Republic of China; 2 Hubei Provincial Center for Disease Control and Prevention, Wuhan, Hubei, People's Republic of China; 3 Wuhan Central Hospital, Wuhan, Hubei, People's Republic of China; 4 Hunan Institute of Parasitic Diseases, Yueyang, Hunan, People's Republic of China; 5 Institute of Geodesy and Geophysics, Chinese Academy of Science, Wuhan, Hubei, People's Republic of China; Centers for Disease Control and Prevention, United States of America

## Abstract

**Introduction:**

More than 80% of schistosomiasis patients in China live in the lake and marshland regions. The purpose of our study is to assess the effect of a comprehensive strategy to control transmission of *Schistosoma japonicum* in marshland regions.

**Methodology/Principal Findings:**

In a cluster randomized controlled trial, we implemented an integrated control strategy in twelve villages from 2009 through 2011 in Gong'an County, Hubei Province. The routine interventions included praziquantel chemotherapy and controlling snails, and were implemented in all villages. New interventions, mainly consisting of building fences to limit the grazing area for bovines, building safe pastures for grazing, improving the residents' health conditions and facilities, were only implemented in six intervention villages. Results showed that the rate of *S. japonicum* infection in humans, bovines, snails, cow dung and mice in the intervention group decreased from 3.41% in 2008 to 0.81% in 2011, 3.3% to none, 11 of 6,219 to none, 3.9% to none and 31.7% to 1.7%, respectively (P<0.001 for all comparisons). In contrast, there were no statistically significant reductions of *S. japonicum* infection in humans, bovines and snails from 2008 to 2011 in the control group (P>0.05 for all comparisons). Moreover, a generalized linear model showed that there was a higher infection risk in humans in the control group than in the intervention group (OR = 1.250, P = 0.001) and an overall significant downward trend in infection risk during the study period.

**Conclusions/Significance:**

The integrated control strategy, designed to reduce the role of bovines and humans as sources of *S. japonicum* infection, was highly effective in controlling the transmission of *S. japonicum* in marshland regions in China.

**Trial Registration:**

Chinese Clinical Trial Registry ChiCTR-PRC-12002405.

## Author Summary

More than 80% of schistosomiasis patients in China live in the lake and marshland regions. Hence, how to control transmission of *Schistosoma japonicum* in these regions is especially important. From 2009 through 2011, we implemented an integrated control strategy, designed to reduce the role of bovines and humans as sources of *S. japonicum* infection, in twelve villages Gong'an County of Hubei Province, which is located in typical marshland. After three years, the rate of *S. japonicum* infection in humans, bovines and snails significantly declined in the six intervention villages. In contrast, there was no significant decline in these indexes in the six control villages. Moreover, there was a higher infection risk in humans in the control group than the intervention group. Our study showed that the integrated control strategy was highly effective in controlling the transmission of *S. japonicum* in marshland regions of China.

## Introduction

Schistosomiasis is an important public health issue in China where it continues to pose a serious threat to human well-being [1]–[3]. Since the People's Republic of China was established in 1949, the Chinese government has given high priority to the control of schistosomiasis, establishing a number of special bodies to manage control activities from the national to the township level [4]–[6]. These policies resulted in remarkable success in that the number of schistosomiasis patients were reduced by over 90%, from about 11.6 million cases in the 1950s to 726,000 cases in 2004 [7]. A further report in 2004 indicated that disease transmission had been interrupted or controlled in 42% of provinces, 40% of counties, and 53% of towns, previously endemic for schistosomiasis [8].

However, prospects for the control of schistosomiasis have been less optimistic in recent years, particularly since the termination of the World Bank Loan Project (WBLP) for schistosomiasis control at the end of 2001 [7]. Compared with the second national survey of *S. japonicum* in China in 1995, the third national survey conducted in 2004 showed that the prevalence of *S. japonicum* infection in humans had not substantially changed in the lake and marshlands and other areas of Southern China [7]. By the end of 2009, a total of 365,770 cases of *S. japonicum* were estimated in China, and 89 counties had not yet reached the criterion for transmission control which stipulated that the human prevalence should be less than 1% over a length of time [9], [10], i.e. the prevalence in these counties was >1%.

Over the past 2–3 decades, the strategies for *S. japonicum* control in southern China, including the vast lake and marshland regions, has involved chemical mollusciciding, alteration of the oncomelanid intermediate snails habitats, and synchronous chemotherapy with praziquantel for all villagers and their bovines [6], [11]. Historically, these strategies achieved some effect, but recent studies demonstrated there were some problems as the control options resulted in environmental pollution leading to ecological damage [11], [12]. Moreover, owing to the high rates of reinfection in both humans and bovines, frequent flooding, and the complex environment, more persons have been infected and the habitat of the *Oncomelania* snails has increased in the lake and marshlands [11], [13]. Consequently, a more effective strategy was needed urgently in these areas.

From 2005 through 2007, an important study of schistosomiasis japonica control was undertaken by Wang et al. in villages along the Poyang Lake in Jiangxi Province involving a comprehensive integrated approach aimed at reducing *S. japonicum* transmission to snails from cattle and humans, which play key roles as sources of *S. japonicum* [14]. The integrated strategy, which included removing bovines from snail-infested grasslands, providing farmers with mechanized farming equipment, building safe water systems, providing adequate sanitation, and implementing health education and synchronous chemotherapy with praziquantel for both villagers and bovines, was highly effective [14].

On the basis of identifying and controlling the main *S. japonicum* infection sources, we implemented a similar strategy (March 2009 through November 2011) to that employed by Wang et al. in the marshlands of Gong'an County [14], Hubei Province, another major endemic area for schistosomiasis japonica. The objective of the study was assessing the strategy's effect in marshland regions.

## Methods

The protocol for this trial and supporting CONSORT checklist are available as supporting information; see Protocol S1 and Checklist S1.

### Ethics statement

Written ethical approval for this study was obtained from the Ethics Review Committee of Hubei Provincial Center for Disease Control and Prevention (no. 200803). Written informed consent was obtained from all adults and from parents or guardians of minors before participation in the study. The participants had the opportunity to withdraw from the study at any time.

Before beginning work on the study, the bovine owners provided consent to have their animals involved in the study. Moreover, permits for the bovines were obtained from Gong'an County Animal Husbandry Bureau. All animal work was carried out under the guidance of the Institute for Laboratory Animal Research (ILAR), and approved by Ethics Review Committee of Animal Experiments, Hubei Provincial Center for Disease Control and Prevention (no. 2008a05).

Both doses of praziquantel (i.e., single 40 mg/kg dose for humans identified as stool egg-positive or 25 mg/kg for infected bovines) were within Chinese Ministry of Health published guidelines [15].

### Study area and participants

Gong'an County is located in typical marshland with a water area of 364 hectares (km^2^) along the mid-to-lower reaches of the Yangtze River in southwest Hubei Province, China. In 2009, there were 294 schistosomiasis-endemic villages (out of a total of 326), 539 cases of advanced schistosomiasis, and 36,612 cases of chronic schistosomiasis in Gong'an County; the prevalence of schistosomiasis in humans and bovines was 2.75% and 2.41%, respectively [16].

**Figure 1:**
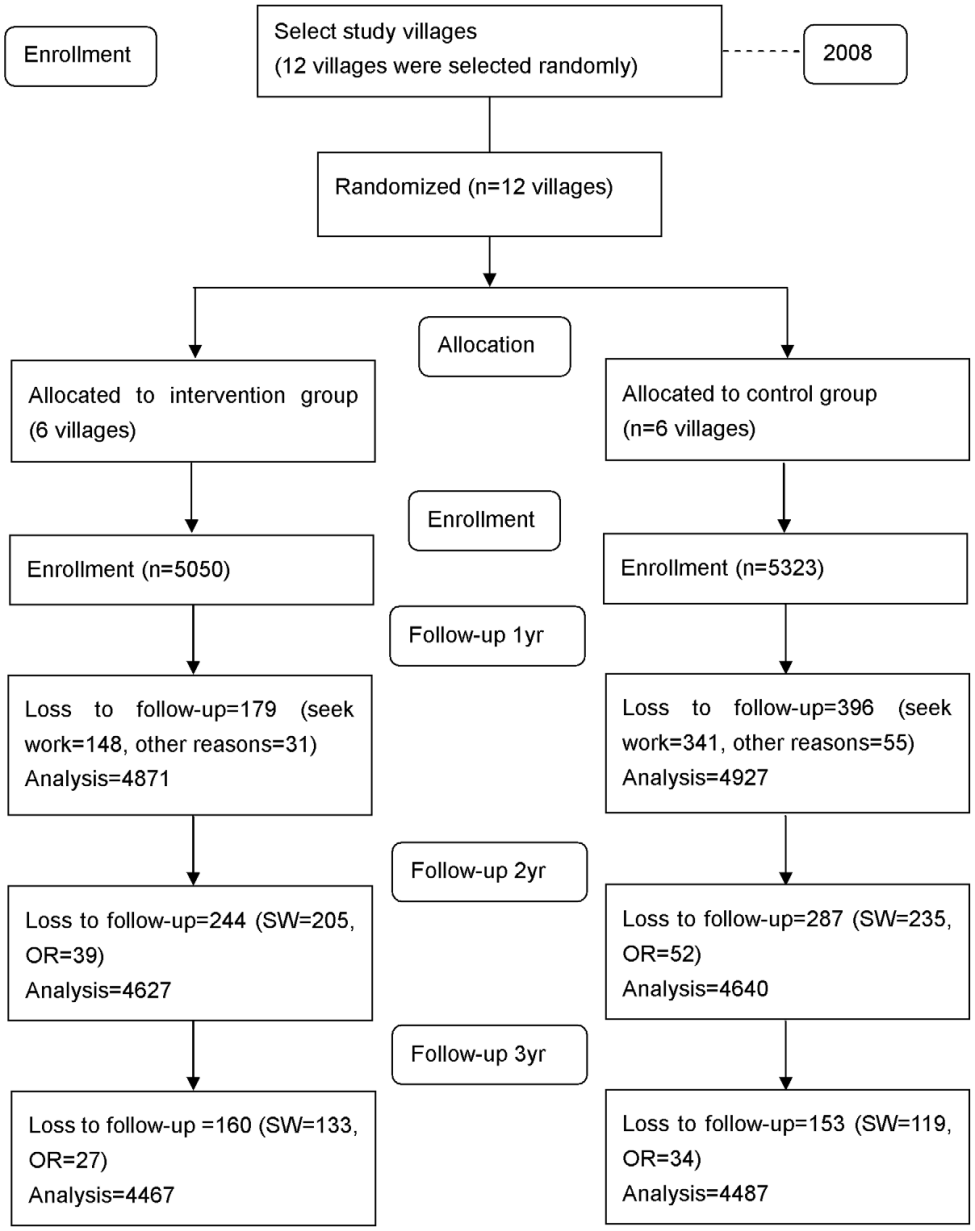
Trial flowchart.

**Figure 2:**
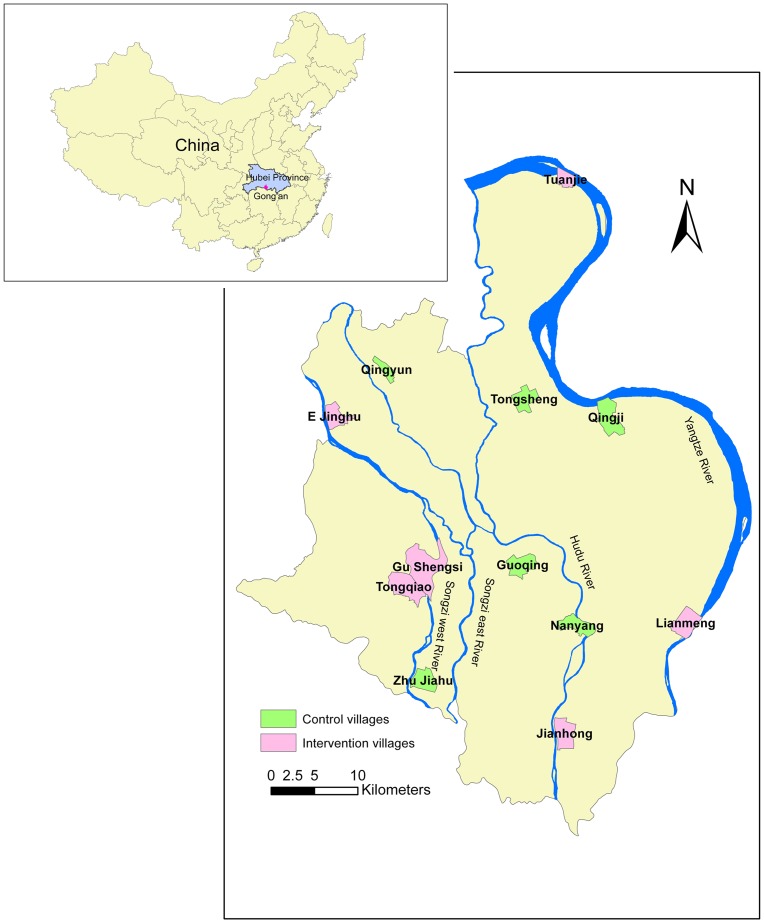
Location of the study villages in the mid-to-lower reaches of the Yangtze River, Hubei province, China.

We carried out the study in 12 villages from 12 towns in Gong'an County which were selected by a two-stage random sampling procedure. The 12 towns were first randomly selected from 16 towns; then 12 schistosomiasis-endemic villages were selected from the 12 towns randomly (each town selected a village). Finally, these villages were randomly divided into intervention and control groups (Figure 1). E Jinghu, Gu Shengsi, Jianhong, Lianmeng, Tongqiao, Tuanjie were assigned to the intervention group and Guoqing, Nanyang, Qingyun, Qingji, Zhu Jiahu, Tongsheng were assigned to the control group (Figure 2). The villages had similar agriculture resources (rice, cotton and brassica napus) and the residents mainly were farmers (about 75%). The residents were exposed to contaminated water when they performed their agricultural or daily activities (i.e. cultivating crops, catching fish and washing clothes).

The participants met the following inclusion criteria: a) must have been a resident of the village for more than 12 months; b) should be aged 6–65 years; c) should continuously reside in the village for the study period; d) have no serious diseases, such as malignant cancer.

### The comprehensive strategy to control transmission of *S. japonicum*

Prior to implementing the new control strategy, routine interventions had been used in Gong'an County to control *S. japonicum* infection, which comprised synchronous praziquantel chemotherapy of both villagers and bovines and mollusciciding of snails [17], [18]. These procedures were continued in all 12 study villages for the duration of the study but additional interventions were incorporated into the intervention group. These comprised of the building of fences to limit the grazing area for bovines, building safe pastures for grazing, improving the residents' health conditions and facilities, and strengthening specialized schistosomiasis clinics at the village level.

#### Fencing and safe pastures

Considering that bovines have been identified as the primary reservoir host of *S. japonicum* infection in China [6], [19]–[21], two measures were implemented (March to May, 2009) to reduce the influence of bovines on transmission. Firstly, 63 kilometres of fencing were installed to prevent animals from grazing in areas where *Oncomelania* snails were present. Warning signs were placed along the fenced areas, with the help of the local government authorities, and professional management staff were employed to ensure the intervention was effective.

Secondly, 7 safe grazing pasture areas were established totaling 190 hectares (1,900,000 m^2^). Each area was 20–30 hectares and was designed to contain about 1,100 animals. The pastures were carefully selected with no or very few snails, which, if present, were quickly eliminated by mollusciciding. Specialized managers were employed to manage each of the grazing areas and ponds were established to enable the bovines to drink uncontaminated water.

Fences comprised cement columns and barbed wire which ensured long-term usage. Each cement column was 160 centimeters (cm) in height and shaped as a triangular prism with 15 cm edges. Each column had three holes through which the barbed wire was fixed, and the distance between each was 2 meters.

#### Improving health and health care facilities

To reduce the potential of humans as a source of infection for snails, measures were undertaken (May to July, 2009) to improve the health and well-being of villagers and the existing health care facilities. Safe water was provided to 2,826 households by building 8 new public wells or improving household wells already in place. To deal with humans feces, 1,545 home lavatories were constructed or repaired with three-cell septic tanks and 618 households were provided with marsh-gas pool latrines. Furthermore, all fishermen were provided with fecal-matter containers to prevent them from excreting feces directly into river or lake freshwater.

#### Strengthening specialized schistosomiasis clinics

In order to implement the interventions in the intervention villages, specialized schistosomiasis clinics were strengthened at the village level (March to May, 2009). As well as being responsible for managing the fences and the safety of the pastures, the clinic staff were responsible for examining the residents and livestock for schistosome infection, treating infected people or livestock with praziquantel, and providing health education on preventing *S. japonicum* infections and, in particular, in explaining the significance of bovines and humans as sources of *S. japonicum* infection for snails.

#### Praziquantel chemotherapy

During the study period, all participants were screened annually for *S. japonicum* infection by indirect hemagglutination assay (IHA), followed by fecal examination of IHA-positives using the Kato-Katz technique [15] (three thick smears). Kato-Katz-positives were treated with praziquantel at a single oral dose of 40 mg/kg. As well, all bovines were checked annually by the miracidium hatching test in dung [15]. All infected bovines were treated with a single dose of praziquantel at a dosage of 25 mg/kg body weight.

#### Snail control

Before implementing the new control strategy, mollusciciding using niclosamide had been conducted in all schistosomiasis-endemic villages (included all 12 study villages) in Gong'an County as part of the routine procedure. During the study period, a comprehensive approach, comprising mollusciciding with niclosamide and environmental modification, which mainly included using cement to change ditches' original environment, was used for snail control in all 12 study villages. Snail surveys were carried out twice-yearly (spring and autumn) along the river banks, ditches and marshlands around the villages [15]. An average of 110 hectares of snail habitat in the study villages were treated annually with molluscicide or environmental modification (89 hectares in 2008, 141 hectares in 2009, 134 hectares in 2010 and 74 hectares in 2011).

### Outcome measures

Five outcome measures, including the primary one (the prevalence of *S. japonicum* in humans) and four secondary ones (the rate of *S. japonicum* infection in bovines, cow dung, snails and mice), were determined annually and used to assess the strategy's effect.

Participants that were positive for both IHA and Kato-Katz were defined as infected and the prevalence of *S. japonicum* of participants in all 12 study villages was in October/November, after the second transmission season.

During the same period, the infection of *S. japonicum* of all bovines was checked in all 12 study villages by the miracidium hatching test in dung [15]. In addition, we gathered fresh cow dung from the grasslands which located in the 6 intervention villages (the sampled locations were the safe grazing pastures from 2009 to 2011), and used the miracidium hatching test to detect the infection of *S. japonicum* in cow dung.

In April and May, we systematically sampled *Oncomelania hupensis* snails along the river banks and in marshlands and ditches around all 12 study villages. In every village, we investigated at least 2000 sample units (0.11 m^2^ frame) and gathered all the live snails in the frame, crushed them, and examined them for *S. japonicum* infection using microscopy.

During the peak of the transmission season, July and August, we used exposure tests with mice to assess the infectivity of water in the 6 intervention villages [14]. Sentinel mice were exposed for 2 hours every day for 3 consecutive days. After 30 to 35 days, we sacrificed the mice and checked for adult worms of *S. japonicum* in their mesenteric veins.

### Statistical analysis

All statistical analyses were performed with the use of Statistics Analysis System, version 9.1 (SAS Institute Inc., Cary, NC, USA). Confidence intervals (CIs) were calculated using standard formulae based on the binomial distribution (annual infection rate of humans). Chi-square test or Fisher exact probability test were used to examine the differences of proportions. A generalized linear model (GLM) with a logit link and a binomial error distribution was used to analyze the risk of *S. japonicum* infection in humans. Generalized estimating equations of parameters with an unstructured variance-covariance matrix were used to account for repeated measures on individuals during the study period. The SAS proc GENMOD was used to estimate the parameters. Two-sided P-values were calculated for all tests and P-values <0.05 were considered statistically significant.

## Results

Table 1 showed the characteristics of the participants in 2008. The differences of age between intervention and control groups were statistically significant (P<0.001).

**Table 1:** Characteristics of the participants in intervention and control groups in 2008.

Characteristics	Control group (n = 5323)	Intervention group (n = 5050)
Age, years	38.4 (13.6)	39.4 (13.4)**
Sex ratio (F/M), n	2686/2637	2617/2433

Values are mean (SD) or number.

** P<0.001 for control group vs. intervention group.

Figure 1 showed the flow diagram of the study design. In total, 10,373 participants were recruited for the study in 2008. The rates of loss to follow-up per year (2009–2011) for each village ranged from 1.1% to 18.5% in the intervention group and 2.1% to 25.2% in the control group (Table 2). The main reason for loss to follow-up was that the participants left the villages to seek work in urban areas.

**Table 2:** *Schistosoma japonicum* infection in humans in intervention and control groups*.

Village	2008			2009			2010			2011		
	N	Infection No.	Prevalence % (CI)	N	Infection No.	Prevalence % (CI)	N	Infection No.	Prevalence % (CI)	N	Infection No.	Prevalence % (CI)
**Intervention group**												
E Jinghu	535	19	3.55 (1.98, 5.12)	508	16	3.15 (1.63, 4.67)	452	10	2.21 (0.85, 3.57)	436	5	1.15 (0.14, 2.15)
Gu Shengsi	1102	38	3.45 (2.37, 4.53)	1054	32	3.04 (2.00, 4.07)	997	19	1.91 (1.06, 2.76)	959	8	0.83 (0.26, 1.41)
Jianhong	765	23	3.01 (1.79, 4.22)	718	17	2.37 (1.25, 3.48)	685	12	1.75 (0.77, 2.74)	637	4	0.63 (0.01, 1.24)
Lianmeng	994	38	3.82 (2.63, 5.02)	964	31	3.22 (2.10, 4.33)	892	19	2.13 (1.18, 3.08)	885	8	0.90 (0.28, 1.53)
Tongqiao	1021	33	3.23 (2.15, 4.32)	1001	24	2.40 (1.45, 3.35)	990	21	2.12 (1.22, 3.02)	963	8	0.83 (0.26, 1.41)
Tuanjie	633	21	3.32 (1.92, 4.72)	626	18	2.88 (1.56, 4.19)	611	12	1.96 (0.86, 3.07)	587	3	0.51 (−0.07, 1.09)
**Total** §	5050	172	3.41 (2.91 ,3.91)	4871	138	2.83 (2.37 ,3.30)	4627	93	2.01 (1.61 ,2.41)	4467	36	0.81 (0.54 ,1.07)
**Control group**												
Guoqing	788	26	3.30 (2.05, 4.55)	755	21	2.78 (1.61, 3.96)	737	22	2.99 (1.75, 4.22)	716	16	2.23 (1.15, 3.32)
Nanyang	1035	37	3.57 (2.44, 4.71)	967	30	3.10 (2.01, 4.20)	921	26	2.82 (1.75, 3.89)	914	23	2.52 (1.50, 3.53)
Qingyun	894	26	2.91 (1.80, 4.01)	812	23	2.83 (1.69, 3.98)	750	21	2.80 (1.62, 3.98)	748	20	2.67 (1.52, 3.83)
Qingji	963	32	3.32 (2.19, 4.46)	889	28	3.15 (2.00, 4.30)	822	25	3.04 (1.86, 4.22)	818	23	2.81 (1.68, 3.95)
Zhu Jiahu	712	22	3.09 (1.82, 4.36)	697	17	2.44 (1.29, 3.59)	683	16	2.34 (1.21, 3.48)	595	16	2.69 (1.39, 3.99)
Tongsheng	931	27	2.90 (1.82, 3.98)	807	22	2.73 (1.60, 3.85)	727	18	2.48 (1.34, 3.61)	696	16	2.30 (1.18, 3.41)
**Total #**	5323	170	3.19 (2.72, 3.67)	4927	141	2.86 (2.40 ,3.33)	4640	128	2.76 (2.29 ,3.23)	4487	114	2.54 (2.08 ,3.00)

* CI denote 95% confidence intervals.

§ As compared with 2008 in the intervention group, there was a statistically significant change in the rate of infection in 2010 and 2011 (P<0.001), and there was no statistically significant change in 2009 (P = 0.101).

# As compared with 2008 in the control group, there was no statistically significant change in the rate of infection from 2009 to 2011 (P = 0.328, P = 0.204, P = 0.055, respectively).

### Infection in humans

The prevalence of *S. japonicum* in humans was 3.41%, 2.83%, 2.01%, and 0.81% from 2008 to 2011 in the intervention villages (comparing with 2008, P = 0.101, P<0.001 and P<0.001, respectively, Table 2). In the control villages, there was no significant statistically decline in the rate of infection from 2008 to 2011 (P>0.05 for all comparisons).

The generalized linear model (Table 3), yielding odds ratios (OR) adjusted for participants' age and gender, showed a higher infection risk in humans in the control villages than the intervention villages (OR = 1.250, P = 0.001); and an overall significant downward trend in infection risk during the study period.

**Table 3:** Analyzing the risk of *Schistosoma japonicum* infection in humans by generalized linear model*.

Variables	OR (95% CI)	P-value
Intercept	0.012 (0.009, 0.016)	<0.001
Age	1.004 (0.999, 1.009)	0.091
Gender		
Female	1.047 (0.919, 1.193)	0.492
Male	1.000	-
Study year		
2008	2.026 (1.670, 2.460)	<0.001
2009	1.734 (1.417, 2.123)	<0.001
2010	1.436 (1.166, 1.768)	<0.001
2011	1.000	-
Group		
Control group	1.250 (1.095, 1.427)	0.001
Intervention group	1.000	-

* The model includes participants' age, gender, study year, group.

### Infection in bovines

The *S. japonicum* infection rates in bovines were 3.3%, 2.9%, 1.3%, and 0.0% from 2008 to 2011 in the intervention villages (comparing with 2008, P = 0.820, P = 0.111 and P<0.001, respectively, Table 4). In contrast, there was no significant decline in the rate of infection during the 4-year period in the control villages (P>0.05 for all comparisons).

**Table 4:** *Schistosoma japonicum* infection in bovines in intervention and control groups*.

Year	Intervention group		Control group				
	N	Infection No. (%)	N	Infection No. (%)	P-value^a^	P-value^b^	P-value^c^
2008	305	10 (3.3)	345	11 (3.2)	-	-	1.000
2009	309	9 (2.9)	337	9 (2.7)	0.820	0.821	1.000
2010	311	4 (1.3)	325	8 (2.5)	0.111	0.646	0.385
2011	318	0 (0.0)	313	8 (2.6)	<0.001	0.650	0.004

* Statistical method was the Fisher exactly probability test.

a Compared with 2008 in the intervention group.

b Compared with 2008 in the control group.

c Compared between the intervention and control groups from 2008 to 2011.

### Infection in snails

From 2008 to 2011, 11 of 6,219, 9 of 5,975, none of 25,010, and none of 15,490 snails were infected with *S. japonicum* in the intervention villages (comparing with 2008, P = 0.824, P<0.001 and P<0.001, respectively, Table 5). In the control villages, there was no significant statistically decline in the rate of infection from 2008 to 2011 (P>0.05 for all comparisons).

**Table 5:** *Schistosoma japonicum* infection in snails in intervention and control groups*.

Year	Intervention group		Control group					
	N	Infection No.	N	Infection No.	P-value^a^	P-value^b^	P-value^c^
2008	6,219	11	4,574	9	-	-	0.824
2009	5,975	9	8,664	6	0.824	0.054	0.187
2010	25,010	0	11,889	13	<0.001	0.231	<0.001
2011	15,490	0	9,493	8	<0.001	0.116	<0.001

* Statistical method was the Fisher exactly probability test.

a Compared with 2008 in the intervention group.

b Compared with 2008 in the control group.

c Compared between the intervention and control groups from 2008 to 2011.

### Infection in cow dung

In the intervention villages in 2008, 21 of 533 (3.9%) of the cow dung samples contained *S. japonicum* eggs. After the implementation of the new control strategy, 15 of 483 (3.1%), 5 of 476 (1.1%) and none of 356 cow dung were infected in 2009, 2010 and 2011, respectively (comparing with 2008, P = 0.501, P = 0.005 and P<0.001, respectively).

### Infectivity of water

In 2008, before implementation of the new control strategy, 19 of 60 (31.7%) mice were infected with *S. japonicum*. In 2009, 2010 and 2011, 13 of 60 mice (21.7%), 7 of 120 (5.8%) and 2 of 120 (1.7%) mice were infected, respectively (comparing with 2008, P = 0.216, P<0.001 and P<0.001, respectively).

### Adverse events

No serious adverse events were reported in the study.

## Discussion

Over the past few years, investigations showed that more than 80% of *S. japonicum* patients in China were found in the lake and marshland areas of Hunan, Hubei, Jiangxi, Anhui, and Jiangsu provinces [22]. Therefore, schistosomiasis control in these regions was especially important.

Because of high rates of *S. japonicum* reinfection in both humans and bovines in marshland and lake regions [21], [23], [24], it was very difficult to reduce the rate of *S. japonicum* infection in humans to a relatively low level (such as less than 1%). The study of Wang et al. showed that a comprehensive control strategy, which was based on interventions to reduce the transmission of *S. japonicum* infection from cattle and humans to snails, can solve this problem [14]. However, the authors mentioned that their study included only a small number of villages and the villages were not selected in a random manner. So they were not sure whether the strategy was still highly effective in other endemic areas. A similar study was implemented in Jiangsu Province of China, but this study did not have parallel control groups [25]. In our study, we applied a similar strategy but strived to avoid these limitations. We adopted a random manner to select 12 villages in Gong'an County, Hubei Province and randomly assigned them to intervention or control groups.

Our study proved that the new comprehensive strategy to control transmission of *S. japonicum* was highly effective in the study areas. Firstly, the prevalence of schistosomiasis in humans declined to a relatively low level after implementing the new interventions. Compared with 2008, the rate of infection in humans in the intervention villages decreased from 3.41% in 2008 to 0.81% in 2011. The generalized linear model showed that a higher infection risk for humans in the control villages than in the intervention villages (OR = 1.250, P = 0.001). Secondly, comparing with 2008, the rate of infection in bovines, snails and cow dung in the intervention group decreased from 3.3% to none, 11 of 6,219 to none, and 3.9% to none in 2011, respectively. Thirdly, during the peak transmission period, after the implementation of the new control strategy, the rate of infection in sentinel mice decreased from 31.7% in 2008 to 1.7% in 2011. It should be noted that the randomized, controlled design pertains only to the human, bovine, and snail data while the environmental cow dung and infectious water data were only collected in the intervention villages.

Bovines are the main infection source of *S. japonicum* in the marshlands and lakes in China, and are responsible for an estimated 75%–90% of the egg contamination [20], [21]. Therefore, it was very important to reduce the role of bovines as an infection source. Recently, the main measures included providing farmers machines instead of bovines and prohibiting bovines in these regions. These measures were effective [14]. However, if these measures were used in some rural areas of Hubei Province, there would be some challenges. Firstly, the machines are not suitable due to the limitation of landform or farmers' knowledge. Secondly, the bovines can bring additional economic benefits for villagers, so the villagers were reluctant to give up cattle rearing.

For these reasons, in our study we adopted new interventions for bovines which included building safe pastures for grazing and preventing bovines from grazing on snail habitat beach areas. Our study proved that these measures were effective. Moreover, these measures were sustainable because the cost of fences and pastures was a one-time expense because the material is durable. We used a triangular cement fence post, which requires less cement than a square shape.

Our study showed that there was no statistically significant decline in the *S. japonicum* infection prevalence in humans, bovines and snails from 2008 to 2011 in the control villages. Past studies showed that the effect of chemotherapy with praziquantel for humans and cattle was limited in controlling schistosomiasis in lake and marshland regions [17]–[19], because it does not prevent reinfection [23]–[25]. In contrast, the integrated control strategy can reduce the transmission of *S. japonicum* infection from humans and cattle to snails, reducing contaminated water as a source of infection in humans [14]. However, this difference is of little practical consequence unless the integrated interventions lead to more sustainable control and a reduction of the basic reproductive number (R_0_). If R_0_ can be kept less than 1 over a length of time, the disease could be gradually eliminated [26]. This is probably the most important potential outcome of the intervention. However, a longer term follow-up study is needed to test this potential outcome. Taken together, we considered that the new interventions, especially the interventions for bovines and humans, were the most important components of the integrated control strategy.

A potential limitation of the study was that we did not investigate the status of *S. japonicum* infection in people who quit the study. Thus, we could not compare the difference of these people's infection between intervention and control groups.

### Conclusions

An integrated control strategy, aiming to weaken the roles of bovines and humans as sources of *S. japonicum* infection, was effective in controlling the transmission of *S. japonicum* in marshland regions of China.

## Supporting Information

Checklist S1 CONSORT Checklist.
(DOC)

Protocol S1 Trial protocol (Chinese language file).
(DOC)
