# Supplementary material for: Assessing the Effect of an Integrated Control Strategy for Schistosomiasis Japonica Emphasizing Bovines in a Marshland Area of Hubei Province, China: A Cluster Randomized Trial
Source: PLoS Negl Trop Dis. 2013 Mar 14;7(3):e2122. doi: 10.1371/journal.pntd.0002122 (PMC3597472; doi:10.1371/journal.pntd.0002122)
Supplement: Protocol S1 — Trial protocol (Chinese language file). (DOC) [file pntd.0002122.s002.doc]

**湖沼地区实施以控制家畜传染源为重点的**

**血吸虫病综合防治策略研究**

**研究方案**

**二 O O 八 年一月**

目 录

[一、前言 1](#__RefHeading___Toc332209554)

[二、总体目标 1](#__RefHeading___Toc332209555)

[三、研究设计 1](#__RefHeading___Toc332209556)

[四、研究地点的选择 1](#__RefHeading___Toc332209557)

[五、研究对象的纳入/排除标准 2](#__RefHeading___Toc332209558)

[六、研究的样本量 2](#__RefHeading___Toc332209559)

[七、干预措施及实施细则 2](#__RefHeading___Toc332209560)

[八、调查内容、重要指标及实施细则 8](#__RefHeading___Toc332209561)

[九、流行病学调查和项目实施情况效果考核 12](#__RefHeading___Toc332209562)

[十、综合评价研究 12](#__RefHeading___Toc332209563)

[十一、计划进度安排 13](#__RefHeading___Toc332209564)

**一、前言**

当前，我国血吸虫病流行最严重的地区主要集中在江湖洲滩，这类地区水位难以控制，杂草丛生，既是天然牧场，又是血吸虫中间宿主钉螺的孳生环境。由于家畜在江湖洲滩敞放，特别是患血吸虫病的家畜动物将带有虫卵的粪便排放于有螺草洲，导致病原扩散并构成人畜易感环境。因此，控制人畜传染源进入有螺洲滩，减少人畜接触疫水，降低人、畜、钉螺血吸虫感染率对预防和控制血吸虫病传播具有重要意义。

**二、总体目标**

探索建立一种以传染源控制为主，既具有良好血防效果、又具有社会经济效益、可持续发展的湖沼型区血吸虫病综合防治模式，降低血吸虫病感染率，取得适合我国国情的湖沼型区血吸虫病防治经验，为后续的推广应用提供科技支撑。

**三、研究设计**

采用整群(以行政村为单位)随机对照试验，干预村实施以传染源控制为主的血吸虫病综合干预策略，对照组采用常规干预策略。整个试验历时4年，从2008年1月1日至2011年12月31日。

**四、研究地点的选择**

本课题的研究现场选择湖北省荆州市公安县。2008年，公安县共有16个乡镇，364个行政村，其中294个行政村是血吸虫病流行村。本研究拟纳入12个流行村，采用两阶段随机整群抽样获得。随机数的产生由统计人员利用SAS程序生成，具体为：第一阶段，把16个乡镇按行政编码编号1至16，采用SAS产生16个随机数（1-16），前12个随机号对应的乡镇即被入选；第二阶段，从12个乡镇随机选择12个流行村，每个乡镇选择一个流行村（排除非流行村），随机方式同第一阶段。最终，将入选的12个流行村编号1至12，并随机等分到干预组和对照组（即每组各6个村）。详细情况见表1。

表1 干预村与对照村地理位置

| 干预组(Intervention villages) | | 对照组(Control villages) | |
| --- | --- | --- | --- |
| 镇名 | 村名 | 镇名 | 村名 |
| 埠河镇 | 团结(Tuanjie) | 斗湖堤镇 | 同升(Tongsheng) |
| 麻豪口镇 | 联盟(Lianmeng) | [杨家厂镇](http://baike.baidu.com/view/1186729.htm) | 青吉(Qingji) |
| 黄山头镇 | 建红(Jianhong) | [毛家港镇](http://baike.baidu.com/view/1186751.htm) | 青云(Qingyun) |
| 章庄铺镇 | 铜桥(Tongqiao) | [章田寺乡](http://baike.baidu.com/view/1186756.htm) | 南阳(Nanyang) |
| 狮子口镇 | 谷升寺(Gu Shengsi) | 孟家溪镇 | 国庆(Guoqing) |
| 斑竹垱镇 | 鹅颈湖(E Jinghu) | 南平镇 | 朱家湖(Zhu Jiahu) |

**五、研究对象的纳入/排除标准**

**1）研究对象纳入标准：**

1、必须是当地常住居民（至少已经住了12个月）；

2、年龄在6至65岁；

3、在研究期间必须住在当地，没有移民（移出村庄）的打算；

4、签署知情同意书。

符合以上四条标准的研究对象均纳入研究。

**2）研究对象排除标准：**

1、排除有精神性、神经系统疾病的患者；

2、排除有严重心、脑、肝、肾等其他严重疾病；

3、排除孕妇或正处于哺乳期的妇女。

符合以上任意一条标准的研究对象均需从研究中剔除。

**特别说明：对于研究对象是人，则需符合以上纳入排除标准；对于研究对象是牛等家畜、采集到的钉螺，原则上均纳入研究**

**3）研究对象纳入起止时间**

对于研究对象是人：从2008年3月1日开始纳入研究对象，截止时间为2008年11月30日。对于研究对象是牛等家畜、钉螺，则从2008年3月1日至2011年11月30日均可以纳入研究。

**六、研究的样本量**

1）研究对象为人：原则上符合条件的常住居民，每个村庄不得低于600人，干预组与对照组样本总量各不得低于5000人，详细情况见调查实施细则。

2）调查对象为牛等家畜和钉螺时，详细情况见调查实施细则。

**七、干预措施及实施细则**

本研究干预措施包括：

1、常规干预措施：病人病畜吡喹酮同步化疗和使用氯硝柳胺药物灭螺，常规干预措施是血吸虫病防控的要求，可严格按照血吸虫病防治手册（第三版）进行，在本方案中不再详述。在本研究中，干预组和对照组均采用了常规干预措施。

2、新干预措施：实施以牛为重点的血吸虫病综合控制策略，包括垸外易感地带围栏封洲隔离带建设和封洲禁牧，建立安全牧场。另外，强化管理和健康教育，包括流行村血防室(卫生室)建设，建立和完善示范区血吸虫病健康教育设施。新的干预措施仅在干预组中实施。

**（一）垸外易感地带围栏封洲隔离带建设和封洲禁牧**

**1、目的**：按照科学规划、流域治理、因地制宜的原则，通过围栏封洲禁牧，采取疏堵结合、强化管理、鼓励土地利用改造洲滩环境等综合治理措施，切断江湖洲滩地区血吸虫病传播途径，达到净化草洲，将江湖洲滩易感环境传播血吸虫病的危害降低到最低程度，控制血吸虫病传播。

**2、垸外易感地带围栏封洲隔离带建设任务**

长度60.0km，辐射7个乡镇27个村垸外洲滩（包括所有的6个干预村，但不包括任意一个对照村）。

表2 湖北省公安县示范区垸外易感地带围栏封洲隔离带建设任务

| **县(市、区)** | **区域** | **辐射范围**  **(行政村)** | **长度**  **(km)** | **完成时间(工作量)** | |
| --- | --- | --- | --- | --- | --- |
|  |  |  |  | 2009年 | 2010年 |
| 湖北省公安县  (合计60.0 km) | 长江荆南外洲 | 1 | 2.0 |  | 2.0 |
|  | 长江外洲 | 2 | 3.5 |  | 3.5 |
|  | 虎渡河外洲 | 6 | 15.0 | 15.0 |  |
|  | 松东河外洲 | 3 | 5.0 |  | 5.0 |
|  | 松西河外洲 | 1 | 2.0 |  | 2.0 |
|  | 危水河外洲 | 10 | 26.0 | 26.0 |  |
|  | 松西河外洲 | 4 | 6.5 |  | 6.5 |
| **合计** | **7个乡镇27处外洲** | **27** | **60.0** | 41.0 | 19.0 |

**3、主要技术要求**

根据不同洲滩地块的大小和地形、地貌特点，在牛羊可能进入的有螺地段，沿易感地带防洪大堤脚边采用水泥桩 + 双股带刺铁丝网、辅以隔离沟对洲滩进行围栏封闭，禁止家畜进入，并防止死角和缺口。

3.1 三棱形水泥柱：用标号42.5号水泥混泥土浇注成边宽12cm×高度1.8m四方形水泥柱，其中埋入地下40cm~50cm，地上部分为1.3m~1.4m，地上部分按30cm~40cm，间隔预留3个小孔，分别用3根5.5mm冷扒丝扎成框架，以便固定双股带刺铁丝。

3.2 水泥柱填埋：每根水泥柱之间间隔为2m， 挖边长为30cm×深30cm~40cm土坑，将水泥柱树立坑内，然后用32.5号水泥卵石混凝土填埋固定，以确保其整体一致性和牢固性。

3.3 双股带刺铁丝安装：选用12号涂有防锈漆的双股带刺铁丝，通过3个预留孔牢固地安装在水泥柱上。

3.4 挖隔离沟：对临水侧外滩较宽的堤段，可在其外侧修建隔离沟，但不得影响堤防安全。隔离沟应平顺规则，上下端与河道(湖泊)贯通，每年淹水时间宜保持连续8个月以上。隔离沟宽度宜采用宽3m～5m，深2m～3m，隔离沟的两侧沟壁应进行坡面硬化。隔离沟坡降比为1:1.2。

3.5 查灭螺处理：对个别局部无法进行围栏的区域必须每年进行查灭螺处理。

**4、工作原则与要求**

4.1各干预村政府对围栏封洲后所辐射的区域制定相应的家畜管理措施，包括封洲禁牧地方法规、乡规民约、公告、告示等，告知疫区群众实施封洲禁牧的意义、地点、时间、管理要求及奖罚规定等，要求群众密切配合和参与。如果是水系相通、草洲相连的乡(镇)或行政村，则采取联防措施，同时发布公告或由上一级发布公告。

4.2 成立工作组、建立责任制，层层落实责任，保证按质按量完成任务。安排专职人员(或血防协管员)进行后期管理和维护，在封洲禁牧期间禁止人、畜进入活动或放牧，建立长效管理机制，确保项目顺利实施。

4.3 确保围栏封洲建设质量，在有条件的地方淘汰耕牛和商品牛，推行以机代牛耕作模式。

4.4 尽可能结合滩地经济开发，探索管理模式，对围栏封洲禁牧区滩地进行植树、种植芦苇或作物种植等经济开发，减少耕牛放牧的滩地，控制耕牛上滩，减少人畜感染血吸虫病机会，达到既利用滩地资源、发展经济，又能实现禁牧以控制家畜传染源、提高社会参与血吸虫病防治的综合效果。

**5、工作目标：**控制人畜传染源进入有螺洲滩，减少人畜接触疫水，降低人、畜、钉螺血吸虫感染率。

**（二）安全牧场建设**

**1、目的**：按照科学规划、因地制宜的原则，通过安全牧场建设，采取疏堵结合、强化管理等措施，实现安全放牧，将江湖洲滩的血吸虫病危害降低到最低程度，控制血吸虫病传播。

**2、建设任务**

湖北省公安县：建设任务为7个，辐射范围为干预组的4个乡镇16个村垸外洲滩（包括所有的6个干预村，但不包括任意一个对照村）。

表3 湖北省公安县示范区安全牧场建设任务

| **县(市、区)** | **区域** | **辐射范围** | **数量** |
| --- | --- | --- | --- |
|  |  |  |  |
| 湖北省公安县  (合计7个) | 长江雷洲外洲 | 1个乡镇3个村 | 2 |
|  | 虎度河外洲 | 1个乡镇3个村 | 2 |
|  | 危水外洲 | 2个乡镇10个村 | 3 |
| **合 计** | 3处外洲 | 4个乡镇16个村 | 7 |

**3、主要技术要求**

**3.1安全牧场选址标准：**在规划建设安全牧场之前，根据放牧动物的种类、数量确定放牧场的范围和规模(每个牧场平均规模*≥*156.25hm^2^)，然后组织有关卫生、农业血防专业技术人员对放牧场地、周边草洲、草坡及水源区螺情进行详细调查，尽可能选择地势较高、常年洪水位淹没不到的无钉螺孳生洲滩。如规划区及周边环境有钉螺孳生，应采取有效灭螺措施进行处理，一般在旱季或秋季水退后在有螺低洼地用氯硝柳胺药浸法或喷洒法消灭钉螺，坑洼地段用机耕平整，将有螺土层埋入地下，播种良种牧草，周边环境采用铲草皮覆盖或硬化，次年3~4月对螺情进行复查复灭，确保牧场安全。

**3.2 牧场建设：**

**3.2.1 建立隔离带：**沿规划牧场四周用水泥柱+双股带刺铁丝将牧场围栏封闭(技术规范按“垸外易感地带围栏封洲隔离带建设”)，在安全牧场出入口处，用红砖水泥建一个长、宽、深为5.0m×3.0m×0.8m消毒池，池内长期保持0.5m深含氯硝柳氨水体，使家畜进出牧场必须通过消毒池，以达到防止尾蚴侵入和杀灭家畜体表尾蚴的作用。选择安全牧场内地势较低的环境，用红砖水泥建一个长、宽、深为10.0m×0.5m×0.5m饮水槽，利用地下渗水以供家畜饮水。

**3.2.2 家畜准入要求：**进入安全牧场内放养的牛羊等家畜，按《动物血吸虫病防治手册》要求定期进行血吸虫病安全检查（尼龙袋集卵顶管孵化法），阳性者按家畜剂量用吡喹酮进行治疗，并于每年3~4月份进行一次扩大化疗，确认无血吸虫感染后方可进入牧场，以达到安全放牧的目的。

**3.2.3 维护与管理：**每个牧场聘请1名懂政策、肯吃苦、责任心强、有一定工作能力的人员担任协管员，对安全牧场进行日常维护和管理，定期对牧场进行查灭螺处理，清洗消毒池、饮水槽和更换池内药物，安全牧场野粪集中、收集与处理，防止人为破坏牧场设施，禁止家畜在牧场外易感地带放牧。

**4、工作原则与要求**

**4.1** 确保安全牧场建设质量，在有条件的地方，改变养殖结构，以养殖家禽如鸡、鸭、鹅等替代养殖家畜。

**4.2** 制定相应的管理措施、建立长效管理机制，在沿洲滩村庄、集镇张贴政府通告，沿堤设置警示宣传牌，向养殖户发放宣传单，组织宣传车宣传安全牧场的意义，让河滩禁牧等政策深入人心，为禁牧工作营造了良好的氛围。安排专职人员(或血防协管员)对安全牧场进行后期管理和维护，对牧场辐射的周边村镇敞养家畜必须进入安全牧场内或安全地带进行放牧。

**4.3**工作目标：解决血吸虫病易感地带围栏封洲禁牧后敞养家畜的安全放牧问题，控制家畜传染源对有螺环境的污染，为周边疫区村的畜牧业发展和家畜饲养创造环境，降低人、畜血吸虫病感染率。

**（三）流行村血防室(卫生室)建设**

**1、建设任务：**在6个干预村分别建设6个流行村血防室(卫生室)， 2009年完成。

2、**主要技术要求**

（1）建设方法：以示范区流行村卫生室为基础，由县血防办指定专人负责村级血防工作的建设，包括对围栏封洲、安全牧场的看管和维护，以及敞养家畜的管理和对畜主的宣传教育，参与人群查治病工作；疫情上报；开展血防健康教育等工作。具体职责为：

①参与组织本村人畜血吸虫病检查和化疗工作。血防专业人员进村开展人畜血吸虫病检查、化疗时，乡村医生利用人熟地熟的条件，协助进行组织发动、收集标本等工作，提高工作效率和查治覆盖率。

②协助血防专业机构人员组织村民查螺、灭螺、汛期灭蚴工作，及时报告螺情。

③配合和协助血防专业人员开展疫情调查和疫情上报工作。乡村医生结合巡诊对接触疫水人员、急性血吸虫病和晚期血吸虫病患者进行排查，及时向血防机构报告急性和晚期血吸虫病疫情。

④向当地居民宣传血防常识，做好健康教育工作。督促所在村学校开展血防宣传教育，配合和协作血防机构人员在易感环境设立警示牌，发放血防宣传资料。宣传栏要用通俗易懂的语言表述，并根据具体情况定期更换其内容，达到宣传和通知的效果，明示当地有血吸虫感染危险的地段，及预防知识等。

⑤承担上级交办的其他事项。

（2）建设要求：①每个血防室必须有完善的工作职责；有规范的疫情报告制度；②建立和规范血防室相关血吸虫病防治资料：在其责任区域内，每年建立有规范的人口分布、人群流动情况、长期或突发性疫情接触情况、家畜饲养情况、人畜血吸虫病发病情况等血吸虫病防治资料；③建立和规范血防室相关血吸虫病防治工作日志；④在村血防室设立1块宣传栏、1个疫情图等。

（3）工作目标：通过乡村血防室建设，进一步健全和完善基层血防工作网络，提高村血防室参与和协助村级血防工作的职责和能力；达到及时了解血吸虫病疫情动态，提升血吸虫病防治和应急处置的能力，建立村血防室、血防乡村医生工作模式。

**（四）完善卫生设施和改善卫生条件**

**1、建设任务：**完善6个试验村的卫生设施和改善卫生条件，建立不少于8个公共饮水井。

2、**主要技术要求**

（1）建设方法：试验村不合格的厕所（粪便没有良好处理手段的厕所），改建为三格化粪池，并鼓励建设沼气化粪池。改善试验村公众饮水井，并在人群集中取水地建设公共饮水井。

**八、调查内容、重要指标及实施细则**

**1、流行村村基本情况调查（附表1～2）**

**调查范围和时段：**调查范围覆盖干预组和对照组所有的12个村庄。以行政村为单位，调查时段从2008年至2011年。

**调查内容：**县(市/区)、乡(镇/场)、村名称与编号、地理位置(GPS经纬度)、流行类型、疫情类型、户数、总人口数、常住人口数、耕地面积（水田、旱田）、改水改厕和安全用水情况、沼气建设情况；年人均GDP、主要经济来源、血吸虫病和其他疾病的年人均医疗费用、村卫生室（血防室）建设状况和参加新农村合作医疗情况。

**2、人群病情调查内容与指标（附表3）**

**调查范围和时段：**调查范围覆盖干预组和对照组所有的12个村庄。以行政村为单位，调查时段从2008年至2011年。2011年后是否需要调查待定。

**调查方法：**对抽样调查村6-65岁常住人口采用血清学检查方法（IHA）进行筛查，血检阳性者采用改良加藤厚涂片法（Kato-Katz法，一粪三片）进行粪检，根据2007年卫生部“血吸虫病病人数调查及推算方案（试行）”计算居民感染率(%)=(血检阳性人数÷血检人数)×(粪检阳性人数÷粪检人数)×100。同时，定性检查其他寄生虫病的感染情况。

**调查内容：**县（市/区）、乡（镇/场）、村名称与编号、当年总人口数、常住人口数、调查对象编号、姓名、性别、年龄、职业、血检与粪检结果、调查人员与调查时间。

**工作要求：**血检受检率不得低于应检人口的90%，粪检受检率不得低于血检阳性人群的90%，应检对象如未能检查，须注明未检原因。

**指标：**血检阳性率、粪检阳性率，居民感染率。

**3、家畜数量及饲养方式调查(附表4)**

**调查范围和时段：**调查范围覆盖干预组和对照组所有的12个村庄。以行政村为单位，调查时段从2008年至2011年。

**调查对象：**调查时段内所有牛羊畜主，包括调查时段内原来饲养过、现在不饲养的畜主。

**调查内容：**县（市/区）、乡（镇/场）、村名称与编号、畜主姓名、所在地垸外洲滩围栏封洲年份、围栏封洲(建安全牧场)前后不同年份的家畜种类、家畜用途、数量、饲养方式及其变化的原因。

**4、家畜病情调查与指标（附表5）**

**调查范围和时段：**调查范围覆盖干预组和对照组所有的12个村庄。以行政村为单位，调查时段从2008年至2011年。

**调查方法：**对抽样调查村牛用塑料杯顶管或孵化瓶粪检法进行查病（一粪三检），受检率不低于应检家畜的95%，不足100头牛的村，检查所有牛只。有羊的村，比照同样方法进行。

**调查内容：**县（市/区）、乡（镇/场）、村名称与编号、畜主姓名与编号、家畜种类、家畜编号、家畜用途、饲养方式、家畜来源、月龄、检查结果。

**工作要求：**一粪三检，受检率不低于应检家畜的95%。

**指标：**粪检阳性率。

**5、家畜敞放情况及洲滩野粪调查(附表6~7)**

**调查范围和时段：**调查范围覆盖干预组和对照组所有的12个村庄。以行政村为单位，调查时段从2008年至2011年。

**调查方法：**每年4～5月和10～11月，在全封洲时期和芦苇收割季节（因为在运输芦苇时，封闭的洲滩可能会开放缺口），分别连续观察2个月的家畜敞放情况和管理情况，同时对人畜常到的有野粪环境，以100m×100m为单元，每处调查2-3个单元的野粪种类、密度，并对新鲜野粪进行孵化或集卵沉淀法检查。

**调查内容：**县（市/区）、乡（镇/场）、村名称与编号、洲滩名称、敞放家畜种类、敞放数量、进入围栏区(在牧场外)的数量、管理方式；野粪调查的区域、环境类型、有螺高程、调查面积、野粪种类、野粪数量、粪检结果等。

**考核指标：**野粪密度、野粪阳性率， 封洲禁牧效果。

**7、垸内外螺情现况调查 (附表8~9)**

**调查范围：**纳入研究的干预组和对照组所有的12个村庄，垸内全部有螺环境、垸外易感地带，按洲滩大小和环境确定，每个洲滩调查框数≥2000框。

**调查时段：**调查时段从2008年至2011年。

**调查方法：**垸外洲滩采取系统抽样法，按20m×20m设框调查；垸内沟渠采用系统抽样结合环境抽查法，按10m等距离设框调查。每框0.11m^2^，捡拾框内所有钉螺（不含钙化螺），解剖镜检，按线、框记录总螺数、活螺数、感染螺数；GPS定位并绘制钉螺分布示意图。

**调查内容：**垸外洲滩面积、垸内外有螺面积、活螺平均密度、活螺框出现率、感染螺平均密度、感染螺框出现率、钉螺感染率、钉螺感染率下降百分比。

**指标：**感染螺平均密度、感染螺框出现率、钉螺感染率、钉螺感染率下降百分比。

**8、调查村查灭螺工作量及消耗调查（附表10）**

**调查范围和时段：**调查范围覆盖干预组和对照组所有的12个村庄。以行政村为单位，调查时段从2008年至2011年。

**调查内容：**县（市/区）、乡（镇/场）、村名称与编号、不同年份的查螺工日、面积和费用，灭螺（蚴）工日、面积和费用，灭螺（蚴）药物用量和费用，化疗药物用量和费用等。

**9、垸外洲滩水体安全性调查(附表11)**

**调查时段：**调查时段从2008年至2011年。

**调查区域：**在建设封洲隔离带与安全牧场的区域，根据洲滩环境和大小，设立垸外水体感染性哨鼠试验监测点。

**监测方法：**在汛期，选择室外温度为25℃左右，晴天或阴天，在人畜易到、水流较为平稳的区域，每个监测点选取30只18～20g的小白鼠，装入铁丝笼内，在水面平稳移动2h，每天感染1次，连续3d，感染后饲养30～35天，解剖观察小白鼠的血吸虫感染情况。饲养过程中死亡的小鼠必须记录死亡时间、数量和解剖观察。

**调查内容：**县（市/区）、乡（镇/场）、村、洲滩名称；感染时间、感染时的气温、水温、感染区域和数量、解剖（死亡）时间、解剖结果（雌虫数、雄虫数、合抱数、阴性鼠数）。

**样本量：**不得少于60只小鼠。

**指标：**感染小鼠数、小鼠感染率、感染虫数。

**10、 综合防治措施调查统计(附表12)**

**调查范围：**调查范围覆盖干预组所有的6个村庄。

**调查内容：**围栏封洲长度与面积、安全牧场处数与面积、健教中心数、村血防室数、圈养畜舍面积、安全用水改造(处\户)、建沼气池数、改厕户数、以机代牛农具数、环改处数、沟渠硬化长度、中小学生健教人次、乡村干部培训人次、血防宣传广播次数、宣传栏、宣传牌、警示牌、封洲禁牧标志牌块数、血防宣传标语数、宣传画册数、血防知识传单、公告、查灭螺、查治病等辐射范围及其成本。

**11、项目建设区成本、效益调查（附表13~14）**

**3.1 调查范围：**调查范围覆盖干预组所有的6个村庄。

**3.2 调查内容：**县（市/区）、乡（镇/场）、村、洲滩（牧场）名称、牧场位置、易感地带长度；建设时间、围栏长度、围栏（牧场）面积、开发种类、开发面积、牧场规模与辐射范围、投入成本（围栏成本、生产成本、人工费用、维护费用）、收益、管理方式。

**指标：**成本-效果指数及成比-效益比。

**九、流行病学调查和项目实施情况效果考核**

1、考核时间：按年度任务进行，每年10～11月。

2、考核内容：

（1）围栏封洲、建立安全牧场等建设任务完成情况、质量与进度；

（2）血吸虫病综合防治措施实施情况；

（3）资料收集和整理情况，检查各附表内容，评价资料收集的质量；

3、考核范围：考核纳入研究的12个流行村。

**十、综合评价研究**

（一）单项指标评价

评价的主要指标：人群血吸虫病感染率。次要指标：牛群血吸虫病感染率、牛粪阳性率、钉螺血吸虫病感染率、水体安全性。评估干预措施的效果，对干预措施的经费投入与产出，进行成本效果与成本效益进行分析与评价。

（二）综合措施效果分析

观察干预组实施以传染源控制为主的综合防治措施后人、畜血吸虫病病情，螺情变化，调查农村社会经济发展和居民增收情况，综合分析和评估示范区实施以控制传染源为主的综合防治措施后示范区血吸虫病防治效果和社会经济发展效益。

（三）建立湖区血吸虫病综合防治模式

通过以传染源控制为主的综合防治措施方案的防治效果和社会经济效益的分析和评估，探索总结既具有良好血防效果、又具有社会经济效益、适合我国新农村建设发展要求，可持续发展的以传染源控制为主的湖区血吸虫病综合防治新模式，新方法。

**十一**、计划进度安排

| **年份** | **研 究 内 容** | **月 份** | | | | | | | | | | | |
| --- | --- | --- | --- | --- | --- | --- | --- | --- | --- | --- | --- | --- | --- |
|  |  | **1** | **2** | **3** | **4** | **5** | **6** | **7** | **8** | **9** | **10** | **11** | **12** |
| **2008** | 选择项目工作现场，制定实施计划 | ★ | ★ | ★ |  |  |  |  |  |  |  |  |  |
|  | 人畜病情流行病学基线调查 |  |  |  | ★ | ★ | ★ | ★ | ★ | ★ | ★ |  |  |
|  | 现有血吸虫病防治措施调查、资料收集 |  |  |  | ★ | ★ | ★ | ★ |  |  |  |  |  |
|  | 垸内外螺情基线调查 |  |  |  | ★ | ★ |  |  |  |  |  |  |  |
|  | 易感地带野粪调查 |  |  |  |  |  |  |  |  | ★ | ★ |  |  |
|  | 垸外洲滩水体感染性基线调查 |  |  |  |  |  | ★ | ★ | ★ | ★ |  |  |  |
| **2009** | 封洲隔离带、安全牧场等建设 |  |  |  |  | ★ | ★ |  |  | ★ | ★ | ★ | ★ |
|  | 垸内外螺情调查 |  |  |  | ★ | ★ |  |  |  |  |  |  |  |
|  | 垸外洲滩水体感染性调查 |  |  |  |  |  | ★ | ★ | ★ | ★ |  |  |  |
|  | 易感地带野粪调查 |  |  |  |  |  |  |  |  | ★ | ★ |  |  |
|  | 人群病情调查、耕牛病情调查 |  |  |  |  |  |  |  |  | ★ | ★ | ★ |  |
|  | 年度资料整理、汇总 |  |  |  |  |  |  |  |  |  |  | ★ | ★ |
| **2010** | 封洲隔离带、安全牧场建设 | ★ | ★ | ★ | ★ | ★ | ★ |  |  |  |  |  |  |
|  | 垸内外螺情调查。 |  |  |  | ★ | ★ |  |  |  |  |  |  |  |
|  | 垸外水体感染性调查 |  |  |  |  |  | ★ | ★ | ★ | ★ |  |  |  |
|  | 易感地带野粪调查 |  |  |  |  |  |  |  |  | ★ | ★ |  |  |
|  | 人群、耕牛病情调查 |  |  |  |  |  |  |  |  | ★ | ★ |  |  |
|  | 年度资料整理、汇总 |  |  |  |  |  |  |  |  |  |  | ★ | ★ |
| **2011** | 垸内外螺情调查 |  |  |  | ★ | ★ |  |  |  |  |  |  |  |
|  | 垸外水体感染性调查 |  |  |  |  |  | ★ | ★ | ★ | ★ |  |  |  |
|  | 易感地带野粪调查 |  |  |  |  |  |  |  |  | ★ | ★ |  |  |
|  | 人群、耕牛病情调查 |  |  |  |  |  |  |  |  | ★ | ★ |  |  |
|  | 年度资料整理、汇总 |  |  |  |  |  |  |  |  |  |  | ★ | ★ |
|  | 资料整理、汇总、分析、  项目评价 |  |  |  |  | ★ | ★ | ★ | ★ |  |  | ★ | ★ |

**附表1 血吸虫病防治项目抽样调查村 年度基本情况调查表**

县(市\区) 乡(镇、场)；县乡编号： 调查时间： 调查人：

| 流行村名称 | 村编号 | GPS位置 | | 流行类型 | 疫情类型 | 总户数 | 总人  口数 | 常住  人口数 | 耕地面积(亩) | | 垸外有螺高程  (m) | 年人均GDP(万元) | 主要经济来源  与比例 | 人均医疗费用（元/人.年） | | 是否有村卫生室(血防室) | 参加农合医疗人数 |
| --- | --- | --- | --- | --- | --- | --- | --- | --- | --- | --- | --- | --- | --- | --- | --- | --- | --- |
|  |  | 经度 | 纬度 |  |  |  |  |  | 水田 | 旱田 |  |  |  | 血吸  虫病 | 其他  疾病 |  |  |
|  |  |  |  |  |  |  |  |  |  |  |  |  |  |  |  |  |  |
|  |  |  |  |  |  |  |  |  |  |  |  |  |  |  |  |  |  |
|  |  |  |  |  |  |  |  |  |  |  |  |  |  |  |  |  |  |
|  |  |  |  |  |  |  |  |  |  |  |  |  |  |  |  |  |  |
|  |  |  |  |  |  |  |  |  |  |  |  |  |  |  |  |  |  |
|  |  |  |  |  |  |  |  |  |  |  |  |  |  |  |  |  |  |
|  |  |  |  |  |  |  |  |  |  |  |  |  |  |  |  |  |  |
|  |  |  |  |  |  |  |  |  |  |  |  |  |  |  |  |  |  |
|  |  |  |  |  |  |  |  |  |  |  |  |  |  |  |  |  |  |
|  |  |  |  |  |  |  |  |  |  |  |  |  |  |  |  |  |  |
|  |  |  |  |  |  |  |  |  |  |  |  |  |  |  |  |  |  |
|  |  |  |  |  |  |  |  |  |  |  |  |  |  |  |  |  |  |
|  |  |  |  |  |  |  |  |  |  |  |  |  |  |  |  |  |  |
|  |  |  |  |  |  |  |  |  |  |  |  |  |  |  |  |  |  |
|  |  |  |  |  |  |  |  |  |  |  |  |  |  |  |  |  |  |
|  |  |  |  |  |  |  |  |  |  |  |  |  |  |  |  |  |  |
|  |  |  |  |  |  |  |  |  |  |  |  |  |  |  |  |  |  |
|  |  |  |  |  |  |  |  |  |  |  |  |  |  |  |  |  |  |

*流行类型：1=洲垸亚型，2=垸内亚型，3=汊滩亚型，4=洲岛亚型，5=水网型， 6=高原平坝型，7=高原峡谷型，8=丘陵型；

*疫情类别：1=一类村，2=二类村，3=三类村，4=四类村，5=五类村； 安全用水：1=自来水，2=井水，3=沟塘水，4=江（河、湖）水，5=泉水

*主要经济来源，有几项填几项：：1=水稻, 2=棉花, 3=苎麻, 4=油菜, 5=畜牧业, 6=水面养殖, 7=外出劳务, 8=其他, 如80%1(估算总收入的比例)

**附表2 血吸虫病防治项目抽样调查村 年度基本情况调查表**

县(市\区) 乡(镇、场)；县乡编号： 调查时间： 调查人：

| 流行村名称 | 村编号 | 已建沼气户数 | 已建三格式卫生厕所 | | 已建沼气池和三格式厕所占农户比例 | 安全用水情况(户数) | | | | 已饮用安全卫生水人数 | 机耕户数 | 牛耕户数 | 机耕和牛耕并用户数 |
| --- | --- | --- | --- | --- | --- | --- | --- | --- | --- | --- | --- | --- | --- |
|  |  |  |  |  |  | 1 | 2 | 3 | 4 |  |  |  |  |
|  |  |  | 座数 | 户数 |  |  |  |  |  |  |  |  |  |
|  |  |  |  |  |  |  |  |  |  |  |  |  |  |
|  |  |  |  |  |  |  |  |  |  |  |  |  |  |
|  |  |  |  |  |  |  |  |  |  |  |  |  |  |
|  |  |  |  |  |  |  |  |  |  |  |  |  |  |
|  |  |  |  |  |  |  |  |  |  |  |  |  |  |
|  |  |  |  |  |  |  |  |  |  |  |  |  |  |
|  |  |  |  |  |  |  |  |  |  |  |  |  |  |
|  |  |  |  |  |  |  |  |  |  |  |  |  |  |
|  |  |  |  |  |  |  |  |  |  |  |  |  |  |
|  |  |  |  |  |  |  |  |  |  |  |  |  |  |
|  |  |  |  |  |  |  |  |  |  |  |  |  |  |
|  |  |  |  |  |  |  |  |  |  |  |  |  |  |
|  |  |  |  |  |  |  |  |  |  |  |  |  |  |
|  |  |  |  |  |  |  |  |  |  |  |  |  |  |
|  |  |  |  |  |  |  |  |  |  |  |  |  |  |
|  |  |  |  |  |  |  |  |  |  |  |  |  |  |
|  |  |  |  |  |  |  |  |  |  |  |  |  |  |
|  |  |  |  |  |  |  |  |  |  |  |  |  |  |
|  |  |  |  |  |  |  |  |  |  |  |  |  |  |
|  |  |  |  |  |  |  |  |  |  |  |  |  |  |

*安全用水：1=自来水， 2=井(压把井)水，3=沟塘水，4=江(河、湖)水

**附表3 血吸虫病防治项目抽样调查村________年度人群病情调查表**

县（市、区） 乡(镇、场) 村 血防站；县乡村编号： 总人口数 常住人口数

| 户号 | 个人号 | 姓名 | 性别 | 年龄 | 职业 | 血检(IHA) | 未检原因 | 粪检片1(Kato-Katz) | | 粪检片2(Kato-Katz) | | 粪检片3(Kato-Katz) | | 粪检孵化 | 未检原因 |
| --- | --- | --- | --- | --- | --- | --- | --- | --- | --- | --- | --- | --- | --- | --- | --- |
|  |  |  |  |  |  |  |  | 血吸虫 | 其他 | 血吸虫 | 其他 | 血吸虫 | 其他 |  |  |
|  |  |  |  |  |  |  |  |  |  |  |  |  |  |  |  |
|  |  |  |  |  |  |  |  |  |  |  |  |  |  |  |  |
|  |  |  |  |  |  |  |  |  |  |  |  |  |  |  |  |
|  |  |  |  |  |  |  |  |  |  |  |  |  |  |  |  |
|  |  |  |  |  |  |  |  |  |  |  |  |  |  |  |  |
|  |  |  |  |  |  |  |  |  |  |  |  |  |  |  |  |
|  |  |  |  |  |  |  |  |  |  |  |  |  |  |  |  |
|  |  |  |  |  |  |  |  |  |  |  |  |  |  |  |  |
|  |  |  |  |  |  |  |  |  |  |  |  |  |  |  |  |
|  |  |  |  |  |  |  |  |  |  |  |  |  |  |  |  |
|  |  |  |  |  |  |  |  |  |  |  |  |  |  |  |  |
|  |  |  |  |  |  |  |  |  |  |  |  |  |  |  |  |
|  |  |  |  |  |  |  |  |  |  |  |  |  |  |  |  |
|  |  |  |  |  |  |  |  |  |  |  |  |  |  |  |  |
|  |  |  |  |  |  |  |  |  |  |  |  |  |  |  |  |
|  |  |  |  |  |  |  |  |  |  |  |  |  |  |  |  |

*性别：男(M)，女(F)； *职业：1农民，2渔船民，3樵民，4家务人员，5学前儿童，6学生，7商业服务，8教师或干部，9其他; *血检结果：阳性(+)，阴性(—)，未检查(9)。

*粪检结果: 阳性(+)，阴性(—)，其他寄生虫卵：1=蛔虫，2=鞭虫，3=钩虫，4=姜片吸虫，5=绦虫，6=其他，并用+，++等表示程度。如1^+++^。阴性(—)，未检查(9)。

*未检查原因：1=外出不在本地，2=不愿意或拒绝，3=检查检查时无粪便送检，4=其他. *粪检孵化：+、—标识阳/阴性.

检验人员： 调查时间

**附表4 血吸虫病防治项目抽样调查村 年度家畜数量与饲养方式调查表**

县（市\区） 乡(镇、场); 县乡编号：

| 村、组名称 | 村编号 | 个人号 | 畜主姓名 | 外洲围栏年份 | 调查年份 | 家畜种类 | 家畜用途 | 数量 | 饲养方式 | 数量变化原因 |
| --- | --- | --- | --- | --- | --- | --- | --- | --- | --- | --- |
|  |  |  |  |  |  |  |  |  |  |  |
|  |  |  |  |  |  |  |  |  |  |  |
|  |  |  |  |  |  |  |  |  |  |  |
|  |  |  |  |  |  |  |  |  |  |  |
|  |  |  |  |  |  |  |  |  |  |  |
|  |  |  |  |  |  |  |  |  |  |  |
|  |  |  |  |  |  |  |  |  |  |  |
|  |  |  |  |  |  |  |  |  |  |  |
|  |  |  |  |  |  |  |  |  |  |  |
|  |  |  |  |  |  |  |  |  |  |  |
|  |  |  |  |  |  |  |  |  |  |  |
|  |  |  |  |  |  |  |  |  |  |  |
|  |  |  |  |  |  |  |  |  |  |  |
|  |  |  |  |  |  |  |  |  |  |  |
|  |  |  |  |  |  |  |  |  |  |  |
|  |  |  |  |  |  |  |  |  |  |  |
|  |  |  |  |  |  |  |  |  |  |  |
|  |  |  |  |  |  |  |  |  |  |  |
|  |  |  |  |  |  |  |  |  |  |  |
|  |  |  |  |  |  |  |  |  |  |  |

*以围栏封洲禁牧前3年开始至本项目完成时止。 *家畜用途：1=肉用，2=耕用，3=肉耕兼用。 *饲养方式：1=敞放洲滩，2=半圈半敞，3=圈养

*数量变化原因：1=封洲后草料减少，无处放牧， 2=草料丰富，洲滩仍可放牧， 3=其他(请简要说明原因)

调查人： 调查时间

**附表5 血吸虫病防治项目抽样调查村________年度家畜粪检调查结果表**

县（市、区） 乡(镇、场) 村 血防站； 县乡村编号：

| 畜主编号 | 畜主姓名 | 家畜种类 | 家畜  编号 | 家畜  用途 | 饲养  方式 | 家畜来源(在对应栏填“是Y”) | | | | 月龄 | 检查结果 | 备注：未检原因等 |
| --- | --- | --- | --- | --- | --- | --- | --- | --- | --- | --- | --- | --- |
|  |  |  |  |  |  | 原有 | 新生 | 买进 | |  |  |  |
|  |  |  |  |  |  |  |  | 疫区 | 非疫区 |  |  |  |
|  |  |  |  |  |  |  |  |  |  |  |  |  |
|  |  |  |  |  |  |  |  |  |  |  |  |  |
|  |  |  |  |  |  |  |  |  |  |  |  |  |
|  |  |  |  |  |  |  |  |  |  |  |  |  |
|  |  |  |  |  |  |  |  |  |  |  |  |  |
|  |  |  |  |  |  |  |  |  |  |  |  |  |
|  |  |  |  |  |  |  |  |  |  |  |  |  |
|  |  |  |  |  |  |  |  |  |  |  |  |  |
|  |  |  |  |  |  |  |  |  |  |  |  |  |
|  |  |  |  |  |  |  |  |  |  |  |  |  |
|  |  |  |  |  |  |  |  |  |  |  |  |  |
|  |  |  |  |  |  |  |  |  |  |  |  |  |
|  |  |  |  |  |  |  |  |  |  |  |  |  |
|  |  |  |  |  |  |  |  |  |  |  |  |  |
|  |  |  |  |  |  |  |  |  |  |  |  |  |
|  |  |  |  |  |  |  |  |  |  |  |  |  |
|  |  |  |  |  |  |  |  |  |  |  |  |  |
|  |  |  |  |  |  |  |  |  |  |  |  |  |

*家畜用途：*家畜用途：1=肉用，2=耕用，3=肉耕兼用。 *饲养方式：1=敞放洲滩，2=半圈半敞，3=圈养。 *检查结果: 用+,++,+++ 等标记感染程度，阴性用“－”记录，未检验者用“9”表示。

*未检查原因: 1=卖出或宰杀， 2=无法获得粪便(包括多头家畜圈养在一起，无法分辨单头粪便)， 3=畜主不愿检查 ，4=其他

检验人员： 调查时间

**附表6 血吸虫病防治项目抽样调查村________年度围栏封洲禁牧区洲滩野粪调查表**

县（市、区） 乡(镇、场)

| 编号(县、乡镇、村号) | 村名 | 洲滩名 | 调查  区域 | 环境类型 | 有螺高程(m) | 调查面积 | | | 野粪种类 | 野粪数量(份) | 野粪孵化结果 |
| --- | --- | --- | --- | --- | --- | --- | --- | --- | --- | --- | --- |
|  |  |  |  |  |  | 长度(m) | 宽度(m) | 面积(千m^2^) |  |  |  |
|  |  |  |  |  |  |  |  |  |  |  |  |
|  |  |  |  |  |  |  |  |  |  |  |  |
|  |  |  |  |  |  |  |  |  |  |  |  |
|  |  |  |  |  |  |  |  |  |  |  |  |
|  |  |  |  |  |  |  |  |  |  |  |  |
|  |  |  |  |  |  |  |  |  |  |  |  |
|  |  |  |  |  |  |  |  |  |  |  |  |
|  |  |  |  |  |  |  |  |  |  |  |  |
|  |  |  |  |  |  |  |  |  |  |  |  |
|  |  |  |  |  |  |  |  |  |  |  |  |
|  |  |  |  |  |  |  |  |  |  |  |  |
|  |  |  |  |  |  |  |  |  |  |  |  |
|  |  |  |  |  |  |  |  |  |  |  |  |
|  |  |  |  |  |  |  |  |  |  |  |  |
|  |  |  |  |  |  |  |  |  |  |  |  |
|  |  |  |  |  |  |  |  |  |  |  |  |
|  |  |  |  |  |  |  |  |  |  |  |  |
|  |  |  |  |  |  |  |  |  |  |  |  |
|  |  |  |  |  |  |  |  |  |  |  |  |
|  |  |  |  |  |  |  |  |  |  |  |  |

*调查区域: A=围栏区内, B=围栏区外, C=牧场外。 *环境类型：1=树林区, 2=芦苇区, 3=低洼湿地, 4=草滩, 5=其他.

*粪检结果: 用+,++,+++,++++ 等符号表示感染程度，阴性结果用“－”记录，未检验者用“9”表示。

调查人员： 调查时间

**附表7 血吸虫病防治项目抽样调查村________年度围栏封洲禁牧区家畜敞放情况调查表**

县（市、区） 乡(镇、场) 村；县乡村编号： 存栏头数：耕牛 菜牛 羊

| 年.月.日 | 敞放家畜  种类 | 敞放  数量 | 进入围栏区  数量 | 在牧场外  敞放数量 | 管理方式 | | | | 发生原因 |
| --- | --- | --- | --- | --- | --- | --- | --- | --- | --- |
|  |  |  |  |  | 赶出围栏区 | 赶入牧场 | 与畜主沟通 | 无人管理 |  |
|  |  |  |  |  |  |  |  |  |  |
|  |  |  |  |  |  |  |  |  |  |
|  |  |  |  |  |  |  |  |  |  |
|  |  |  |  |  |  |  |  |  |  |
|  |  |  |  |  |  |  |  |  |  |
|  |  |  |  |  |  |  |  |  |  |
|  |  |  |  |  |  |  |  |  |  |
|  |  |  |  |  |  |  |  |  |  |
|  |  |  |  |  |  |  |  |  |  |
|  |  |  |  |  |  |  |  |  |  |
|  |  |  |  |  |  |  |  |  |  |
|  |  |  |  |  |  |  |  |  |  |
|  |  |  |  |  |  |  |  |  |  |
|  |  |  |  |  |  |  |  |  |  |
|  |  |  |  |  |  |  |  |  |  |
|  |  |  |  |  |  |  |  |  |  |
|  |  |  |  |  |  |  |  |  |  |
|  |  |  |  |  |  |  |  |  |  |
|  |  |  |  |  |  |  |  |  |  |

*注释：管理方式直接在选择栏打√，不需要填写者保留空格； *发生原因：填写家畜为什么进入围栏内，或者没有在安全牧场放养的原因，如围栏损坏、芦苇收割季节围栏开出口，牧场辐射范围小，畜主不合作等，简要填写。

调查人员： 调查时间

**附表8 血吸虫病防治示范区项目覆盖村垸内螺情调查表**

县(市\区) 乡(镇、场)；县乡编号： 调查时间： 调查人：

| 流行村  名称 | 村组编号 | 调查  年份 | 环境名称 | 环境类型 | 植被种类 | 调查面积(万m^2^) | 有螺面积(万m^2^) | 调查  框数（框） | 有螺  框数（框） | 总螺数（只） | 活螺数（只） | 解剖螺数（只） | 阳性螺  框数(只) | 阳性  螺数（只） | 阳性螺面积(万m^2^) | 阳性螺处数（处） | 钉螺感染率(%) |
| --- | --- | --- | --- | --- | --- | --- | --- | --- | --- | --- | --- | --- | --- | --- | --- | --- | --- |
|  |  |  |  |  |  |  |  |  |  |  |  |  |  |  |  |  |  |
|  |  |  |  |  |  |  |  |  |  |  |  |  |  |  |  |  |  |
|  |  |  |  |  |  |  |  |  |  |  |  |  |  |  |  |  |  |
|  |  |  |  |  |  |  |  |  |  |  |  |  |  |  |  |  |  |
|  |  |  |  |  |  |  |  |  |  |  |  |  |  |  |  |  |  |
|  |  |  |  |  |  |  |  |  |  |  |  |  |  |  |  |  |  |
|  |  |  |  |  |  |  |  |  |  |  |  |  |  |  |  |  |  |
|  |  |  |  |  |  |  |  |  |  |  |  |  |  |  |  |  |  |
|  |  |  |  |  |  |  |  |  |  |  |  |  |  |  |  |  |  |
|  |  |  |  |  |  |  |  |  |  |  |  |  |  |  |  |  |  |
|  |  |  |  |  |  |  |  |  |  |  |  |  |  |  |  |  |  |
|  |  |  |  |  |  |  |  |  |  |  |  |  |  |  |  |  |  |
|  |  |  |  |  |  |  |  |  |  |  |  |  |  |  |  |  |  |
|  |  |  |  |  |  |  |  |  |  |  |  |  |  |  |  |  |  |
|  |  |  |  |  |  |  |  |  |  |  |  |  |  |  |  |  |  |
|  |  |  |  |  |  |  |  |  |  |  |  |  |  |  |  |  |  |
|  |  |  |  |  |  |  |  |  |  |  |  |  |  |  |  |  |  |
|  |  |  |  |  |  |  |  |  |  |  |  |  |  |  |  |  |  |
|  |  |  |  |  |  |  |  |  |  |  |  |  |  |  |  |  |  |

*环境类型：1=河流，2=沟渠，3=水田，4=塘堰，5=旱地，6=江滩，7=洲滩，8=其他； *植被种类：1=杂草，2=芦苇，3=树林，4=水稻，5=其他。 *框数单位：0.11m^2^/框

**附表9 血吸虫病防治示范区项目覆盖村垸外螺情调查表**

县(市\区) 乡(镇、场)；县乡编号： 调查时间： 调查人：

| 流行村名称 | 村编号 | 调查  年份 | 环境名称 | 环境类型 | 植被种类 | 洲滩面积(万m^2^) | 易感地带 | | 围栏年份 | 围栏长度(m) | 围栏面积(万m^2^) | 调查面积(万m^2^) | 有螺面积(万m^2^) | 调查  框数(框) | 有螺  框数（框） | 总螺数（只） | 活螺数(只) | 解剖螺数（只） | 阳性螺  框数（框） | 阳性  螺数（只） | 阳性螺面积(万m^2^) | 钉螺感染率(%) |
| --- | --- | --- | --- | --- | --- | --- | --- | --- | --- | --- | --- | --- | --- | --- | --- | --- | --- | --- | --- | --- | --- | --- |
|  |  |  |  |  |  |  | 长度(m) | 面积(万m^2^) |  |  |  |  |  |  |  |  |  |  |  |  |  |  |
|  |  |  |  |  |  |  |  |  |  |  |  |  |  |  |  |  |  |  |  |  |  |  |
|  |  |  |  |  |  |  |  |  |  |  |  |  |  |  |  |  |  |  |  |  |  |  |
|  |  |  |  |  |  |  |  |  |  |  |  |  |  |  |  |  |  |  |  |  |  |  |
|  |  |  |  |  |  |  |  |  |  |  |  |  |  |  |  |  |  |  |  |  |  |  |
|  |  |  |  |  |  |  |  |  |  |  |  |  |  |  |  |  |  |  |  |  |  |  |
|  |  |  |  |  |  |  |  |  |  |  |  |  |  |  |  |  |  |  |  |  |  |  |
|  |  |  |  |  |  |  |  |  |  |  |  |  |  |  |  |  |  |  |  |  |  |  |
|  |  |  |  |  |  |  |  |  |  |  |  |  |  |  |  |  |  |  |  |  |  |  |
|  |  |  |  |  |  |  |  |  |  |  |  |  |  |  |  |  |  |  |  |  |  |  |
|  |  |  |  |  |  |  |  |  |  |  |  |  |  |  |  |  |  |  |  |  |  |  |
|  |  |  |  |  |  |  |  |  |  |  |  |  |  |  |  |  |  |  |  |  |  |  |
|  |  |  |  |  |  |  |  |  |  |  |  |  |  |  |  |  |  |  |  |  |  |  |
|  |  |  |  |  |  |  |  |  |  |  |  |  |  |  |  |  |  |  |  |  |  |  |
|  |  |  |  |  |  |  |  |  |  |  |  |  |  |  |  |  |  |  |  |  |  |  |
|  |  |  |  |  |  |  |  |  |  |  |  |  |  |  |  |  |  |  |  |  |  |  |
|  |  |  |  |  |  |  |  |  |  |  |  |  |  |  |  |  |  |  |  |  |  |  |
|  |  |  |  |  |  |  |  |  |  |  |  |  |  |  |  |  |  |  |  |  |  |  |

*环境类型：1=河流，2=沟渠，3=水田，4=塘堰，5=旱地，6=江滩，7=洲滩，8=其他； *植被种类：1=杂草，2=芦苇，3=树林，4=水稻，5=其他。 *框数单位：0.11m^2^/框

*提供各洲滩GPS位点数据图

**附表10 血吸虫病防治项目样调查村 年度查灭螺工作量及消耗调查表**

县（市\区） 乡(镇、场); 县乡编号：

| 流行村名称 | 村编号 | 调查年份 | 查螺工作统计 | | | | (1、药物 2、工程) 灭螺(蚴)工作统计 | | | | | | 灭螺药物量(kg) | 灭蚴药物量(kg) | 查螺  费用(元) | 灭螺(蚴)  费用(元) | 吡喹酮用量(片) | | 化疗费用(元) |
| --- | --- | --- | --- | --- | --- | --- | --- | --- | --- | --- | --- | --- | --- | --- | --- | --- | --- | --- | --- |
|  |  |  | 工日 | | 面积(万m^2^) | | 工日 | | | 面积(万m^2^) | | |  |  |  |  |  |  |  |
|  |  |  |  |  |  |  |  |  |  |  |  |  |  |  |  |  | 人 | 畜 |  |
|  |  |  | 垸内 | 垸外 | 垸内 | 垸外 | 垸内 | 垸外 | 灭蚴 | 垸内 | 垸外 | 灭蚴 |  |  |  |  |  |  |  |
|  |  |  |  |  |  |  |  |  |  |  |  |  |  |  |  |  |  |  |  |
|  |  |  |  |  |  |  |  |  |  |  |  |  |  |  |  |  |  |  |  |
|  |  |  |  |  |  |  |  |  |  |  |  |  |  |  |  |  |  |  |  |
|  |  |  |  |  |  |  |  |  |  |  |  |  |  |  |  |  |  |  |  |
|  |  |  |  |  |  |  |  |  |  |  |  |  |  |  |  |  |  |  |  |
|  |  |  |  |  |  |  |  |  |  |  |  |  |  |  |  |  |  |  |  |
|  |  |  |  |  |  |  |  |  |  |  |  |  |  |  |  |  |  |  |  |
|  |  |  |  |  |  |  |  |  |  |  |  |  |  |  |  |  |  |  |  |
|  |  |  |  |  |  |  |  |  |  |  |  |  |  |  |  |  |  |  |  |
|  |  |  |  |  |  |  |  |  |  |  |  |  |  |  |  |  |  |  |  |
|  |  |  |  |  |  |  |  |  |  |  |  |  |  |  |  |  |  |  |  |
|  |  |  |  |  |  |  |  |  |  |  |  |  |  |  |  |  |  |  |  |
|  |  |  |  |  |  |  |  |  |  |  |  |  |  |  |  |  |  |  |  |
|  |  |  |  |  |  |  |  |  |  |  |  |  |  |  |  |  |  |  |  |
|  |  |  |  |  |  |  |  |  |  |  |  |  |  |  |  |  |  |  |  |
|  |  |  |  |  |  |  |  |  |  |  |  |  |  |  |  |  |  |  |  |
|  |  |  |  |  |  |  |  |  |  |  |  |  |  |  |  |  |  |  |  |
|  |  |  |  |  |  |  |  |  |  |  |  |  |  |  |  |  |  |  |  |
|  |  |  |  |  |  |  |  |  |  |  |  |  |  |  |  |  |  |  |  |

*调查年份：以围栏封洲禁牧前3年开始至本项目完成时止。

**附表11 血吸虫病防治示范区________年围栏封洲禁牧区(安全牧场区)内外哨鼠监测表**

县（市、区） 乡(镇、场) 村； 县乡村编号：

| 感染情况 | | | | | | 检查情况 | | | | | | |
| --- | --- | --- | --- | --- | --- | --- | --- | --- | --- | --- | --- | --- |
| 感染时间 | 洲滩名 | 气温(℃) | 水温(℃) | 感染区域 | 哨鼠数量 | 解剖(死亡)时间 | 感染区域 | 序号 | 雌虫数 | 雌虫数 | 合抱数 | 阴性 |
|  |  |  |  |  |  |  |  |  |  |  |  |  |
|  |  |  |  |  |  |  |  |  |  |  |  |  |
|  |  |  |  |  |  |  |  |  |  |  |  |  |
|  |  |  |  |  |  |  |  |  |  |  |  |  |
|  |  |  |  |  |  |  |  |  |  |  |  |  |
|  |  |  |  |  |  |  |  |  |  |  |  |  |
|  |  |  |  |  |  |  |  |  |  |  |  |  |
|  |  |  |  |  |  |  |  |  |  |  |  |  |
|  |  |  |  |  |  |  |  |  |  |  |  |  |
|  |  |  |  |  |  |  |  |  |  |  |  |  |
|  |  |  |  |  |  |  |  |  |  |  |  |  |
|  |  |  |  |  |  |  |  |  |  |  |  |  |
|  |  |  |  |  |  |  |  |  |  |  |  |  |
|  |  |  |  |  |  |  |  |  |  |  |  |  |
|  |  |  |  |  |  |  |  |  |  |  |  |  |
|  |  |  |  |  |  |  |  |  |  |  |  |  |
|  |  |  |  |  |  |  |  |  |  |  |  |  |
|  |  |  |  |  |  |  |  |  |  |  |  |  |
|  |  |  |  |  |  |  |  |  |  |  |  |  |

*感染区域：A=围栏内， B=围栏外(堤脚区)， C=牧场内， D=牧场外

检验人员： 监测时间

**附表12 血吸虫病项目抽样村 年度综合防治措施调查表**

**县（市、区）**

| 防治措施  建设时间 | 建设时间段 | 数量 | | 辐射区域 | | | | 投入成本  (万元) | 经费来源 | 建设内容 |
| --- | --- | --- | --- | --- | --- | --- | --- | --- | --- | --- |
|  |  | 历史 | 项目期内 | 乡镇数 | 村数 | 人口数 | 牛羊数量 |  |  |  |
| 围栏封洲(长度km，面积-万m^2^) |  |  |  |  |  |  |  |  |  |  |
| 安全牧场处数、面积(万m^2^) |  |  |  |  |  |  |  |  |  |  |
| 健教中心(个) |  |  |  |  |  |  |  |  |  |  |
| 村血防室(个) |  |  |  |  |  |  |  |  |  |  |
| 圈养畜舍(面积) |  |  |  |  |  |  |  |  |  |  |
| 安全用水改造(处\户) |  |  |  |  |  |  |  |  |  |  |
| 建沼气池数 |  |  |  |  |  |  |  |  |  |  |
| 改厕户数 |  |  |  |  |  |  |  |  |  |  |
| 农机具数(以机代牛) |  |  |  |  |  |  |  |  |  |  |
| 环改处数 |  |  |  |  |  |  |  |  |  |  |
| 沟渠硬化(km) |  |  |  |  |  |  |  |  |  |  |
| 中小学生健教课(人次) |  |  |  |  |  |  |  |  |  |  |
| 乡村干部培训(人次) |  |  |  |  |  |  |  |  |  |  |
| 广播(次) |  |  |  |  |  |  |  |  |  |  |
| 宣传栏(个) |  |  |  |  |  |  |  |  |  |  |
| 宣传牌(个) |  |  |  |  |  |  |  |  |  |  |
| 警示牌(个) |  |  |  |  |  |  |  |  |  |  |
| 封洲禁牧标志(块) |  |  |  |  |  |  |  |  |  |  |
| 标语(条) |  |  |  |  |  |  |  |  |  |  |
| 宣传画册(张/册) |  |  |  |  |  |  |  |  |  |  |
| 传单(张) |  |  |  |  |  |  |  |  |  |  |
| 公告(条) |  |  |  |  |  |  |  |  |  |  |
| 查灭螺 |  |  |  |  |  |  |  |  |  |  |
| 查治病 |  |  |  |  |  |  |  |  |  |  |

其他：

**附表13 血吸虫病防治示范区成本、效果调查表**

**县（市、区） 金额单位：万元**

| 建设  时间 | 洲滩位置 | | 洲滩名称 | 易感地带长度(km) | 围栏长度(km) | 围栏面积(万m^2^) | 开发种类 | 开发面积(万m^2^) | 总成本 | 围栏成本 | | 生产成本 | | 维护  费用 | 收入 | 管理  方式 |
| --- | --- | --- | --- | --- | --- | --- | --- | --- | --- | --- | --- | --- | --- | --- | --- | --- |
|  | 乡镇名 | 村名 |  |  |  |  |  |  |  | 材料 | 人工 | 材料 | 人工 |  |  |  |
|  |  |  |  |  |  |  |  |  |  |  |  |  |  |  |  |  |
|  |  |  |  |  |  |  |  |  |  |  |  |  |  |  |  |  |
|  |  |  |  |  |  |  |  |  |  |  |  |  |  |  |  |  |
|  |  |  |  |  |  |  |  |  |  |  |  |  |  |  |  |  |
|  |  |  |  |  |  |  |  |  |  |  |  |  |  |  |  |  |
|  |  |  |  |  |  |  |  |  |  |  |  |  |  |  |  |  |
|  |  |  |  |  |  |  |  |  |  |  |  |  |  |  |  |  |
|  |  |  |  |  |  |  |  |  |  |  |  |  |  |  |  |  |
|  |  |  |  |  |  |  |  |  |  |  |  |  |  |  |  |  |
|  |  |  |  |  |  |  |  |  |  |  |  |  |  |  |  |  |
|  |  |  |  |  |  |  |  |  |  |  |  |  |  |  |  |  |
|  |  |  |  |  |  |  |  |  |  |  |  |  |  |  |  |  |
|  |  |  |  |  |  |  |  |  |  |  |  |  |  |  |  |  |
|  |  |  |  |  |  |  |  |  |  |  |  |  |  |  |  |  |
|  |  |  |  |  |  |  |  |  |  |  |  |  |  |  |  |  |
|  |  |  |  |  |  |  |  |  |  |  |  |  |  |  |  |  |
|  |  |  |  |  |  |  |  |  |  |  |  |  |  |  |  |  |
|  |  |  |  |  |  |  |  |  |  |  |  |  |  |  |  |  |
|  |  |  |  |  |  |  |  |  |  |  |  |  |  |  |  |  |

*开发种类：A=芦苇，B=植树，C=其他(请注明) *总成本=围栏+开发各项成本； *芦苇按年成本和年收入计算，植树按10年投入成本和预期(实际)收入计算；

*管理方式：1=个人承包经营， 2=集体经营。

产业调整变化：

调查人员： 调查时间

**附表14 血吸虫病防治示范区成本、效果调查表**

**县（市、区） 金额单位：万元**

| 建设  时间 | 牧场位置 | | 牧场名称 | 易感地带长度(km) | 牧场面积(亩) | 容纳家畜数 | 辐射范围 | | | | | 总成本 | 建设材料成本 | 人工  费用 | 年维护费用 | 年收入 | |
| --- | --- | --- | --- | --- | --- | --- | --- | --- | --- | --- | --- | --- | --- | --- | --- | --- | --- |
|  | 乡 | 村 |  |  |  |  | 乡数 | 村数 | 人口数 | 牛数 | 羊数 |  |  |  |  | 来源 | 金额 |
|  |  |  |  |  |  |  |  |  |  |  |  |  |  |  |  |  |  |
|  |  |  |  |  |  |  |  |  |  |  |  |  |  |  |  |  |  |
|  |  |  |  |  |  |  |  |  |  |  |  |  |  |  |  |  |  |
|  |  |  |  |  |  |  |  |  |  |  |  |  |  |  |  |  |  |
|  |  |  |  |  |  |  |  |  |  |  |  |  |  |  |  |  |  |
|  |  |  |  |  |  |  |  |  |  |  |  |  |  |  |  |  |  |
|  |  |  |  |  |  |  |  |  |  |  |  |  |  |  |  |  |  |
|  |  |  |  |  |  |  |  |  |  |  |  |  |  |  |  |  |  |
|  |  |  |  |  |  |  |  |  |  |  |  |  |  |  |  |  |  |
|  |  |  |  |  |  |  |  |  |  |  |  |  |  |  |  |  |  |
|  |  |  |  |  |  |  |  |  |  |  |  |  |  |  |  |  |  |
|  |  |  |  |  |  |  |  |  |  |  |  |  |  |  |  |  |  |
|  |  |  |  |  |  |  |  |  |  |  |  |  |  |  |  |  |  |
|  |  |  |  |  |  |  |  |  |  |  |  |  |  |  |  |  |  |
|  |  |  |  |  |  |  |  |  |  |  |  |  |  |  |  |  |  |
|  |  |  |  |  |  |  |  |  |  |  |  |  |  |  |  |  |  |
|  |  |  |  |  |  |  |  |  |  |  |  |  |  |  |  |  |  |
|  |  |  |  |  |  |  |  |  |  |  |  |  |  |  |  |  |  |
|  |  |  |  |  |  |  |  |  |  |  |  |  |  |  |  |  |  |
|  |  |  |  |  |  |  |  |  |  |  |  |  |  |  |  |  |  |

管理方式：

调查人员： 调查时间
